# Extensive transmission of SARS-CoV-2 BQ.1* variant in a population with high levels of hybrid immunity: A prevalence survey

**DOI:** 10.1016/j.ijid.2023.11.039

**Published:** 2024-02

**Authors:** Juan P. Aguilar Ticona, Meng Xiao, Dan Li, Nivison Nery, Matt Hitchings, Emilia M. M. Andrade Belitardo, Mariam O. Fofana, Renato Victoriano, Jaqueline S. Cruz, Laise de Moraes, Icaro Morais Strobel, Jessica Jesus Silva, Ananias Sena do Aragão Filho, Guilherme S. Ribeiro, Mitermayer G. Reis, Federico Costa, Ricardo Khouri, Albert I. Ko, Derek A.T. Cummings

**Affiliations:** 1 Instituto de Saúde Coletiva, Universidade Federal da Bahia, Salvador, Brazil; 2 Instituto Gonçalo Moniz, Fundação Oswaldo Cruz, Ministério da Saúde, Salvador, Brazil; 3 Department of Epidemiology of Microbial Diseases, Yale School of Public Health, New Haven, United States; 4 Department of Laboratory Medicine, State Key Laboratory of Complex Severe and Rare Diseases, Peking Union Medical College Hospital, Chinese Academy of Medical Sciences and Peking Union Medical College, Beijing, China; 5 Public Health Emergency Center, Chinese Center for Disease Control and Prevention, Beijing, China; 6 Department of Biostatistics, University of Florida, Gainesville, United States; 7 Faculdade de Medicina da Bahia, Universidade Federal da Bahia, Salvador, Brazil; 8 Department of Biology, University of Florida, Gainesville, United States; 9 Emerging Pathogens Institute, University of Florida, Gainesville, United States

**Keywords:** SARS-CoV-2, BQ.1 variant, High incidence, Hybrid immunity

## Abstract

**Objectives:**

The SARS-CoV-2 BQ.1* variant rapidly spread globally in late 2022, posing a challenge due to its increased immune evasion.

**Methods:**

We conducted a prevalence survey in Brazil from November 16 to December 22, 2022, as part of a cohort study. We conducted interviews and collected nasal samples for reverse transcription-polymerase chain reaction (RT-PCR) testing and whole-genome sequencing. Cumulative incidence was estimated using RT-PCR positivity, cycle threshold values, and external data on the dynamics of RT-PCR positivity following infection.

**Results:**

Among 535 participants, 54% had documented SARS-CoV-2 exposure before this outbreak and 74% had received COVID-19 vaccination. In this study, 14.8% tested positive for SARS-CoV-2, with BQ.1* identified in 90.7% of cases. Using case data and cycle threshold values, cumulative incidence was estimated at 56% (95% confidence interval, 36-88%). Of the 79 positive participants, 48.1% had a symptomatic illness, with a lower proportion fulfilling the World Health Organization COVID-19 case definition compared to prior Omicron waves. No participants required medical attention.

**Conclusions:**

Despite high population-level hybrid immunity, the BQ.1* variant attacked 56% of our population. Lower disease severity was associated with BQ.1* compared to prior Omicron variants. Hybrid immunity may provide protection against future SARS-CoV-2 variants but in this case was not able to prevent widespread transmission.

## Highlights

• We found a high incidence (56%) of BQ.1 in a community with high hybrid immunity.
• BQ.1 exhibits a different pattern of milder symptoms compared to previous variants.
• We used a new methodology that uses viral load to estimate the incidence.

## Introduction

The Omicron variant of SARS-CoV-2 has been characterized by high levels of immune evasion [1]. The most recently emerged subvariants, BQ.1.1 and XBB, have been shown to effectively evade immunity generated by vaccines, including bivalent formulations designed specifically to target Omicron BA.5 [1], [2], [3]. In addition to diminishing vaccine effectiveness, the continued evolution of Omicron variants may limit the utility of available treatment options such as Nirmatrelvir/ritonavir or molnupiravir [4,5]. Moreover, changes in the clinical spectrum of disease may result in biased estimates of transmission from symptom-based surveillance [6,7].

Laboratory studies have identified mutations that confer twice as much immune evasion in BQ.1 and BQ.1.1 subvariants (here referred to collectively as BQ.1*) compared to the BA.4 and BA.5 subvariants [8]. However, it remains unknown how much BQ.1* associated immune evasion affects transmission among populations with pre-existing immunity, especially those with hybrid immunity (immunity due to exposure to both infection and vaccination). Prior studies of transmission during the circulation of the Omicron BA.1 subvariant demonstrated a high incidence of reinfection and breakthrough infections among vaccinated individuals, and the degree of protection conferred by prior infection and vaccination is known to decline over time [9], [10], [11].

This study aims to estimate the incidence of polymerase chain reaction (PCR)-confirmed infection with the SARS-CoV-2 Omicron BQ.1* subvariant in a population in Salvador, Brazil, with a high prevalence of hybrid immunity. We performed a population-based prevalence survey of SARS-CoV-2 infection using molecular diagnostics and whole-genome sequencing and applied novel computational approaches to infer the incidence of infection using the distribution of PCR cycle threshold (Ct) values [12]. We also used the Ct values of samples to gain broader insights on BQ.1* transmission. We compared the severity of illness associated with BQ.1* infection to other Omicron variants, estimated household secondary attack rate, and examined risk factors associated with the acquisition of infection.

## Methods

### Setting and study design

**Figure 1:**
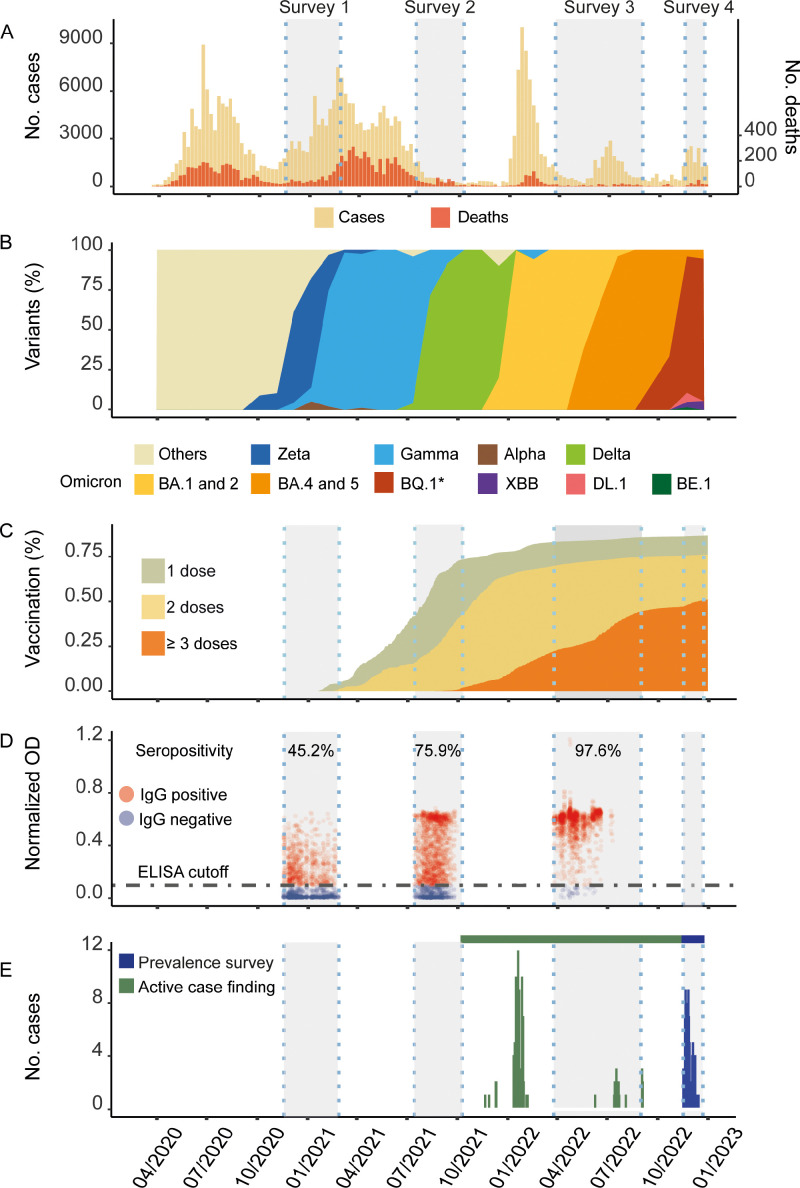
COVID-19 pandemic in Salvador and in the study site. (a) Weekly number of SARS-CoV-2 cases and deaths in Salvador, Brazil. (b) Distribution of SARS-CoV-2 subvariants in Salvador (c) Cumulative proportion of COVID-19 vaccination dose administered amongst Salvador residents. (d) SARS-CoV-2 IgG testing results in previous seroprevalence surveys in recruited individuals. Red dots: SARS-CoV-2 IgG; blue dots: SARS-CoV-2 IgG negative; horizontal grey dot-dash line: OD cut-off value of 0.5). (e) Number of SARS-CoV-2 cases identified in Pau da Lima. ELISA, enzyme-linked immunosorbent assay; Ig, immunoglobulin.

This study was conducted in Salvador, the capital of the state of Bahia, Brazil, which has experienced five major COVID-19 waves since early 2020 (Figure 1a), with the three most recent waves in 2022 driven by Omicron subvariants (Figure 1b). The first Omicron wave occurred from January to March 2022, mainly attributed to BA.1* and BA.2* subvariants, while the second wave, from June to September 2022, was attributed to BA.4* and BA.5*. The third wave in November 2022 was predominantly due to the BQ.1* subvariant (Figure 1b). By November 2022, 86% of Salvador's residents had received at least one dose of a COVID-19 vaccine, and 75% had received at least two doses (Figure 1c).

In this context, we conducted a population-based prevalence survey in Pau da Lima, a slum community in Salvador. This community is in an area of 0.17 km^2^. Approximately 85% of inhabitants were squatters without legal title to their homes, and 50% had a per capita household income of less than $1.25 per day. In 2003, an open cohort study was initiated in the area to investigate infectious diseases including leptospirosis and arbovirus infections, with bi-annual or annual follow-ups. After 2020, the study's scope was expanded to include COVID-19 studies [13]. Three serosurveys conducted from November 2020 to August 2022 (Surveys 1-3) showed an increase in seropositivity (tested by SARS-CoV-2 anti-S immunoglobulin G) among participants (Figure 1d). An active case-finding study between November 2021 and October 2022 identified symptomatic SARS-CoV-2 cases and their contacts in the same area (Figure 1e). During the active case-finding study, our field teams visited study households every 2 weeks to screen residents for symptoms and collect nasal swabs for SARS-CoV-2 molecular diagnostics.

The results of this work are part of the fourth COVID-19 survey conducted in the cohort between November 16 and December 22, 2022. During this period, Salvador, as well as the Pau da Lima community, experienced a high increase in the number of cases associated with the transmission of the SARS-CoV-2 variant BQ.1.

### Participants and study procedures

We included individuals aged 2 years or older who slept at least 3 nights per week within the study area and provided consent to participate. Field technicians performed data collection, including interviews and collecting biological samples. After obtaining informed consent, a standardized questionnaire was administered to collect sociodemographic information (age, sex, schooling, self-reported ethnicity, and income), COVID-19 symptoms, and vaccination history. Symptomatic individuals were defined as those who reported any of the following symptoms in the week preceding or during the visit: fever, cough, fatigue, headache, myalgia, sore throat, congestion or runny nose, dyspnea, nausea, diarrhea, anorexia, loss of taste, loss of smell or mental state altered [14]. Each participant provided an anterior nasal swab for SARS-CoV-2 molecular testing, and symptomatic cases and their household contacts were administered a rapid antigen test during the initial visit. Positive cases were immediately informed, and healthcare assistance recommendations were given.

### Laboratory examination of SARS-Cov-2 infection

Real-time reverse transcription-PCR (RT-PCR) was conducted to confirm SARS-CoV-2 infection, and the PCR Ct values for the ORF1ab gene were recorded for positive samples. Next-generation sequencing (NGS) using the Illumina method was performed on positive samples to identify variants of concern (VOCs) and/or variants of interest (VOIs). Both molecular diagnostic tests were conducted by the COVID Platform of Fiocruz-Bahia, Brazil. For phylogenetic analysis, Omicron lineage sequences collected during both the active case-finding and prevalence survey periods from Pau da Lima were compared with SARS-CoV-2 Omicron variant data from Salvador, Brazil, obtained from the GISAID database between January 01, 2022, and December 31, 2022 (see details in Supplementary Material 1).

### Data analysis: descriptive analyses

To describe the characteristics of the study participants, we used absolute frequencies and percentages for categorical variables and median and interquartile range (IQR) for numeric variables. We compared continuous variables with Mann-Whitney U and categorical variables with Fisher's exact test or chi-square test as appropriate, and linear by linear chi-square tests for ordinal categorical variables. Statistical analysis was performed using R Statistical Software version 3.1.6.

### Data analysis: estimation of cumulative incidence

We used a method for estimating the epidemic growth rate of SARS-CoV-2 using RT-PCR Ct values, as previously described by Hay et al. [12]. Briefly, the daily prevalence of RT-PCR positivity together with the Ct values among RT-PCR-positive samples was used to estimate the daily probability of infection. To ensure we used only tests that represented a random sample of individuals with respect to infection risk, we excluded tests collected at the day 7 follow-up visit. To inform the distribution of Ct after infection, we used published data on Omicron infections (see details in Supplementary Material 1). The assumed Ct distribution over time since infection was consistent with the observed Ct over time from symptom onset observed in symptomatic individuals in our population (Supplementary Figure 3). We estimated the overall cumulative incidence of infection from October 19, 2022 to December 22, 2022. Based on the available literature, the probability of testing RT-PCR positive 28 days after Omicron infection is small [15,16], meaning that the Ct values we measured provided no information about incidence before October 19. Using the estimated incidence over time, we estimated the day of peak incidence, as well as RT-PCR positivity prevalence by week to assess goodness of fit.

We conducted sensitivity analyses to compare the recruited and nonrecruited participants, to determine the robustness of our sample to identify PCR-positive participants in our cohort that were used to estimate the incidence. Also, we performed sensitivity analyses to check the robustness of the cumulative incidence estimate to changes in the CT distribution and PCR positivity probability over time (Supplementary Material 1).

### Data analysis: symptom evaluation

We compared the frequency of reported symptoms and medical attention between participants identified through active case finding during a period dominated by BA.1 and BA.5 variants, and those recruited in the current survey. Symptomatic cases were identified based on any symptom associated with COVID-19, as mentioned previously in the text. Then, we proceeded to evaluate the proportion of infections meeting World Health Organization (WHO)’s definition of a symptomatic case, which includes an acute onset of fever and cough, or three or more of the following symptoms: fever, cough, weakness/fatigue, headache, myalgia, sore throat, coryza, dyspnea, nausea, diarrhea, and anorexia [14].

### Data analysis: secondary attack rate

To estimate the secondary attack rate (SAR), we defined the index case as the individual with the earliest positive COVID-19 test or symptom onset. Co-index cases were two or more household members who tested positive or had symptom onset on the same date. One co-index case was selected randomly as the index case to calculate the SAR. Household contacts were individuals who lived in the same household as the index case within 7 days after the positive PCR test result or onset of symptoms. A secondary case was a household contact who tested positive for SARS-CoV-2. The SAR was calculated by dividing the number of secondary cases by the total number of non-index household residents. Additionally, using the imputed datasets generated to evaluate the sample selection, we estimated the SAR for the entire cohort and compared it with the observed result.

### Secondary data resource

To describe the context of the SARS-CoV-2 transmission in Salvador, we used data on daily infections and deaths in Salvador and the Pau da Lima sanitary district since the beginning of the pandemic from the Brazil Ministry of Health (https://covid.saude.gov.br) and the Center for Strategic Information for Health Surveillance (CIEVS) (http://www.cievs.saude.salvador.ba.gov.br/), respectively. The prevalence of SARS-CoV-2 variants in Salvador over time was obtained from the Fiocruz COVID-19 Genomic Surveillance Network (https://pvm-igm.github.io), while data on vaccination were obtained from the Brazil Ministry of Health (https://opendatasus.saude.gov.br/).

## Results

### Participants characteristics

We surveyed 293 households, totaling 929 residents, with 535 meeting the inclusion criteria and participating in the study by completing questionnaires and providing biological samples. The remaining 378 residents were excluded due to reasons such as moving out, absence during visits, or declining to participate. Additionally, 16 residents were excluded due to invalid PCR results (Supplementary Figure 1).

The sociodemographic characteristics of the participants are presented in Table 1 according to the SARS-CoV-2 immunological status. Briefly, 57.9% (310/535) were female, and the median age was 32 years (IQR 16-47 years). 49.0% (262/535) self-identified as black, and 46.7% (250/535) reported an income below the international poverty line (US$2.15 per person per day). Overall, 95.8% (518/535) of participants have received at least one dose of a COVID-19 vaccine or have a history of SARS-CoV-2 infection.

**Table 1:** Demographic and SARS-CoV-2 immunological characteristics of participants, Salvador, Brazil.

Characteristics|	No. (%)		*P*-value
	SARS-CoV-2 positive n = 79	SARS-CoV-2 negative n = 456	
Sex			0.426
Female	49 (62.0)	261 (57.2)	
Male	30 (38.0)	195 (42.8)	
Age group, year			0.941
<18	24 (30.4)	126 (27.6)	
18-35	21 (26.6)	131 (28.7)	
36-59	26 (32.9)	147 (32.2)	
≥60	8 (10.1)	52 (11.4)	
Ethnicity^a^			0.384
Black	35 (45.5)	227 (50.0)	
Brown	35 (45.5)	203 (44.7)	
Other	7 (9.1)	24 (5.3)	
Education^a^			0.104
Never studied	7 (8.9)	19 (4.2)	
Primary and middle school	51 (64.6)	276 (60.9)	
High school and higher	21 (26.6)	158 (34.9)	
Income category			0.105
<US$ 2.15/day	42 (53.2)	208 (45.6)	
US$ 2.15-3.63/day	16 (20.3)	84 (18.4)	
>US$ 3.63/day	21 (26.6)	164 (36.0)	
Prior vaccination			0.516
≥3 doses	42 (53.2)	220 (48.2)	
2 doses	17 (21.5)	124 (27.2)	
1 dose	11 (13.9)	36 (7.9)	
0 dose	9 (11.4)	76 (16.7)	
Prior documented SARS-CoV-2 exposure			0.004
Yes^b^	31 (39.2)	258 (56.6)	
No	48 (60.8)	198 (43.4)	
Prior documented SARS-CoV-2 exposure and vaccination^c^			0.032
Yes	24 (30.4)	197 (43.2)	
No	55 (69.6)	259 (56.8)	
Prior documented SARS-CoV-2 exposure or vaccination^d^			>0.999
Yes	77 (97.5)	441 (96.7)	
No	2 (2.5)	15 (3.3)	

a There were two and three individuals having missing values of their ethnicity and education, respectively, in the SARS-CoV-2 negative group.

b A SARS-CoV-2 seroconversion observed before the first dose of vaccination, or previous molecular confirmed infection during active case finding.

c "Yes" indicates individuals with ≥1 dose of vaccination and evidence of prior exposure at the same time; "No" indicates individuals without prior vaccination or without evidence of prior exposure.

d "Yes" indicates individuals with ≥1 dose of vaccination or evidence of prior exposure; "No" indicates individuals without vaccination and without having evidence of prior exposure.

### Crude and variant-specific prevalence

**Figure 2:**
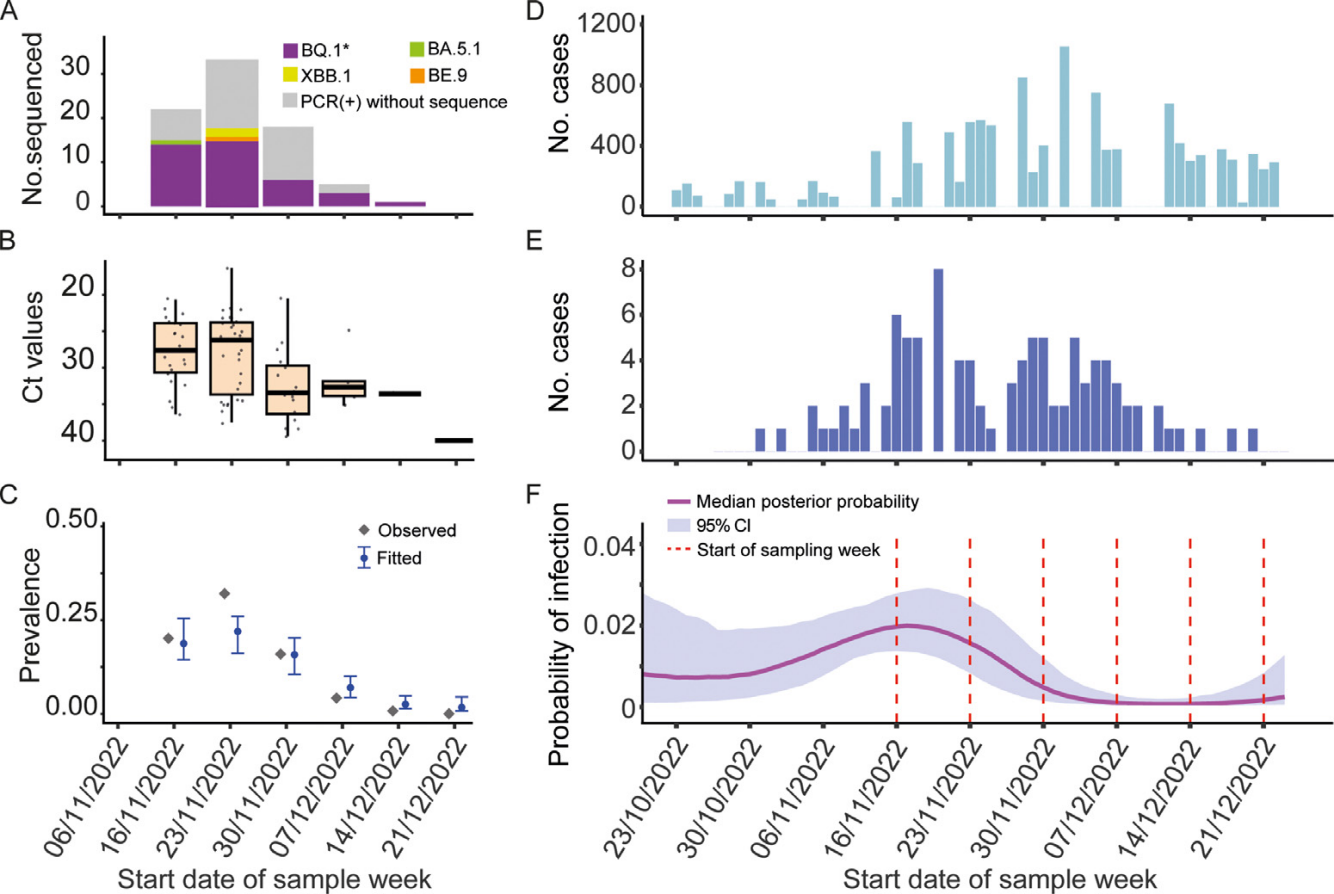
Characterizations of the BQ.1* wave. (a) Number of different subvariant amongst molecular testing positive individuals. (b) Ct value of SARS-CoV-2 cases grouped by week. (c) Weekly observed prevalence (grey diamonds) and fitted median prevalence with 95% CI (blue points and error bars). (d) SARS-CoV-2 daily cases reported in Salvador. (e) SARS-CoV-2 daily cases reported in Pau da Lima sanitary district. (f) Median posterior trajectory for the incidence curve. CI, confidence interval; Ct, cycle threshold; PCR, polymerase chain reaction.

A total of 79 cases of SARS-CoV-2 were identified, with an overall crude prevalence of 14.8% (95% confidence interval [CI] 11.8-17.8%) (Supplementary Figure 1). Among 58 positive RT-PCR samples analyzed using NGS, 15 cases (25.9%) could not be classified at the subvariant level due to low genome coverage (<70%). Among the remaining 43 cases, BQ.1* was detected in 39 cases (90.7%). Of these, 30 cases (69.8%) were BQ.1.1, eight cases (18.6%) were BQ.1, and one case (2.3%) was BQ.1.22. The BA.5.1 and BE.9 Omicron subvariants each accounted for one case (2.3%), while two cases (4.7%) of XBB.1 were identified during the second week of sample collection (Figure 2a).

**Figure 3:**
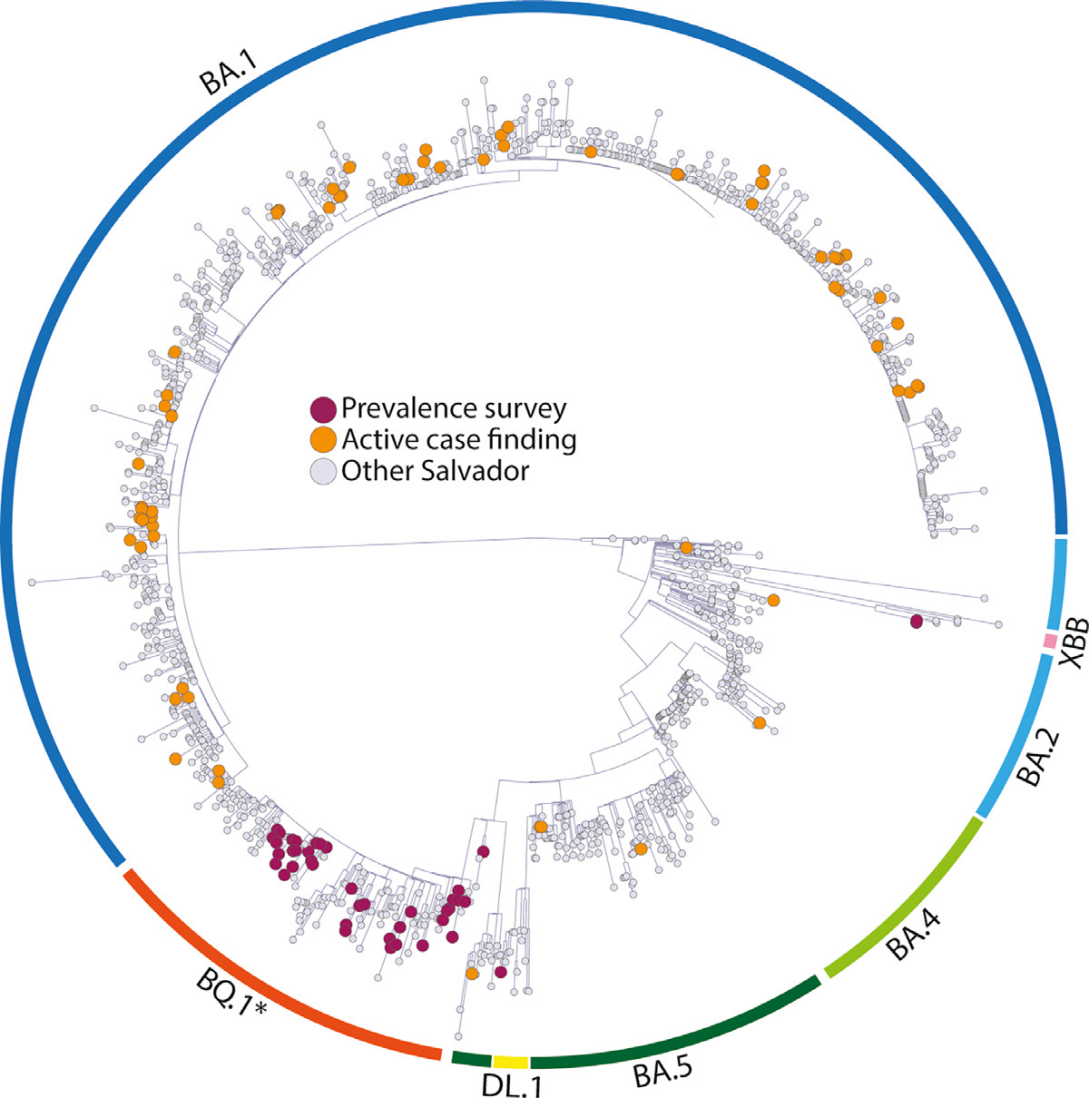
Genome-based phylogenetic tree of SARS-CoV-2 Omicron subvariants identified in this study and in the city of Salvador, Brazil.

Phylogenetic analysis included 1263 samples from Salvador, 88 from the previous active case-finding period, and 43 from the present survey. Viruses from Pau da Lima and Salvador were closely related, and no genetic clustering within these two geographic areas was identified. Like Pau da Lima, the circulation of XBB in Salvador was lower than that of BQ.1 during the study period (Figure 3).

### Prevalence over time and cumulative incidence

Figure 2b shows the distribution of Ct values in SARS-CoV-2 RT-PCR-positive samples by epidemiological week. Lower Ct values were observed in the first 2 weeks, with a subsequent increase in the following 3 weeks. These changes matched the observed weekly SARS-CoV-2 prevalence trends, which peaked at 32.1% in the second week (November 23-29, 2022) before gradually decreasing (Figure 2c). The prevalence trends in the study population were consistent with data from the Pau da Lima sanitary district, with a two-week lag for the peak of the BQ.1 wave compared to Salvador's overall peak (Figures 2d and 2e).

The estimated cumulative incidence of infection from October 19 to December 22, 2022, was 56% (95% credible interval [CrI] = 36 to 88%), with the peak incidence on November 17th (95% CrI = 9th to 21st) during the first sampling week (Figure 2f). Due to the lag between incident infections and viral clearance, the peak of the estimated incidence curve appeared earlier than the peak of observed prevalence (Figures 2e and 2f). The overall RT-PCR positivity was well-fitted by the model (11.7% vs observed 12.4% among individuals swabbed at the initial household visit), but the peak prevalence in week 47 was underestimated (22.0% vs observed 32.1%) (Figure 2c).

In additional analyses, using multiple imputations to account for missing data and estimate the proportion of participants who were PCR-positive (Supplementary Table 4-6), our findings remained unchanged. In sensitivity analysis varying key features of the assumed Ct distribution over time following infection, the estimated cumulative incidence ranged from 49% to 62% (Supplementary Table 7).

### Clinical symptoms and medical attention after infection

Clinical symptoms were assessed in 38 (48.1%, 95% CI = 37.1-59.1) SARS-CoV-2-positive symptomatic individuals during the BQ.1* wave and compared to 103 positive cases from prior Omicron waves. Rhinorrhea was the most frequently reported symptom during the BQ.1* wave (78.9%), followed by cough, headache, and sore throat, each reported by more than 50% of participants (Table 2). The number of symptoms reported was similar between the BQ.1* wave and previous waves. However, individuals in the BQ.1* wave were more likely to report shortness of breath (47.4% vs 14.6%, *P* <0.001) and less likely to report diarrhea (2.6% vs 16.5%, *P* = 0.043) (Table 2). Additionally, the proportion of symptomatic cases meeting the WHO definition criteria was significantly lower during the BQ.1* wave compared to previous waves (47.4% vs 69.9%, *P* = 0.023) (Table 2). None of the SARS-CoV-2-positive individuals during the BQ.1* wave required medical attention, in contrast to 3.8% (95% CI = 1.2-9.1%) during previous waves (Table 2).

**Table 2:** Symptoms and severity outcomes of symptomatic SARS-CoV-2 positive participants during the BQ.1 wave vs in previous omicron waves, Salvor, Brazil.

Characteristics	No. (%) or median (IQR)	*P*-value	
	SARS-CoV-2 positive in the BQ.1 survey^a^ n = 38	SARS-CoV-2 positive in the active case finding^a^ n = 103	
No. of symptoms	4.0 (2.0-6.0)	4.0 (2.5-6.5)	0.590
Frequency of symptoms			
Rhinorrhea	30 (78.9)	70 (68.0)	0.287
Cough	24 (63.2)	78 (75.7)	0.205
Headache	19 (50.0)	64 (62.1)	0.268
Sore throat	19 (50.0)	58 (56.3)	0.633
Short of breath	18 (47.4)	15 (14.6)	<0.001
Fever	14 (36.8)	52 (50.5)	0.211
Fatigue	10 (26.3)	26 (25.2)	1
Shiver	7 (18.4)	20 (19.4)	1
Myalgia	6 (15.8)	29 (28.2)	0.198
Anorexia	4 (10.5)	15 (14.6)	0.781
Loss of taste	4 (10.5)	10 (9.7)	1
Loss of smell	3 (7.9)	8 (7.8)	1
Diarrhea	1 (2.6)	17 (16.5)	0.043
Nausea	1 (2.6)	12 (11.7)	0.186
Mental state altered	1 (2.6)	2 (1.9)	1
Other symptoms^b^	4 (10.5)	7 (6.8)	0.705
Meet the World Health Organization COVID-19 case definition^c^	18 (47.4)	72 (69.9)	0.023
Healthcare need			
Medical attention, n (%)	0 (0)	4 (3.8)	0.567
Urgent care visit, n (%)	0 (0)	3 (2.7)	0.773
Hospitalization, n (%)	0 (0)	0 (0)	NA

a BQ.1 survey was conducted between November 16 and December 22, 2022 and the active case finding was conducted between November 20, 2021, to October 26, 202.

b Other symptoms, besides at least one mentioned in the list, included eye discomfort, knuckle, abdominal, chest or lower back pain, itching, and bitterness in the mouth.

c World Health Organization definition: acute onset of fever and cough, or acute onset of any three or more of the following signs or symptoms: fever, cough, general weakness/fatigue, headache, myalgia, sore throat, coryza, dyspnea, nausea, diarrhea and anorexia.

### Household secondary attack rate

Among 54 households with at least one confirmed case of SARS-CoV-2, we selected 115 residents from 35 households with more than one resident to estimate the secondary attack rate (SAR). Among these participants, 35 were classified as index cases, 25 were secondary cases, and 55 were negative contacts (Supplementary Figure 2). The crude SAR was 31.3% (95% CI = 22.2-42.1), and other SARs stratified by non-index characteristics are presented in Supplementary Table 1. Individuals under 18 were more likely to be secondary cases compared to those 18 and older (relative risk = 2.03, 95% CI = 1.04-3.95). Using multiple imputations to estimate the SAR in the entire cohort while considering the household number of residents distribution, sex, age, vaccination, and previous participation in the previous survey, we found a low SAR in households with a high number of residents (Supplementary Figure 4). However, the 95% CIs from the observed and estimated data overlap in both the pooled SAR and the stratified analysis by household number of residents (Supplementary Figure 4 and Supplementary Table 8).

### Documented prior exposure

A detailed description of the evolution of seroprevalence and vaccination in the cohort before the outbreak described here, aimed at understanding hybrid immunity in this community, is provided in Supplementary Table 2. However, due to uncertainties regarding seropositivity associated with vaccination or infection, and the loss of follow-up, it was not possible to clearly define prior exposure associated with either or both once vaccination became available (after survey 1). The evaluation of risk factors associated with BQ.1 PCR positivity is outlined in Supplementary Table 3. We identified a signal of protection (odds ratio = 0.50; 95% CI = 0.25-0.97) suggesting that previous infection during survey 1, conducted from November 2020 to February 2021, may serve as a proxy for a potentially lower risk of reinfection during the subsequent months until the BQ.1* outbreak in this community.

## Discussion

We describe a rapid and large outbreak predominantly caused by BQ.1* that we estimated affected 56% (95% CrI = 36 to 88%) of individuals in our population over 5 weeks. Our population was previously highly exposed with 97% having detectable immunity to SARS-CoV-2 from prior infection and/or high rates of vaccination before the outbreak we describe here. Our findings highlight that even populations in which a high proportion of individuals have been previously infected and/or vaccinated can experience substantial outbreaks of BQ.1* [17,18]. During the study period, BQ.1* was the most prevalent variant (90.7%) compared to XBB. This differs from other regions such as Singapore and India [19], [20], [21], [22] where XBB emerged as the most common variant at the end of 2022. While BQ.1 remained the predominant variant in the US and Europe until the last weeks of 2022, increasing trends of XBB have been observed in these regions. In the first and sixth week of 2023, XBB became the most prevalent variant in the US and Europe, respectively [22,23], while the incidence of XBB in Brazil remained low. The mechanisms driving the emergence of one strain over the other are not understood [23].

Although this population had a high incidence of infection, medically attended illness rates were extremely low. Compared to a previous period of BA.1 predominance, fewer individuals met WHO clinical diagnosis criteria during the BQ.1* wave. This change in symptom presentation may lead to an underestimation of BQ.1* incidence from surveillance based on clinical criteria. Similar shifts in symptom patterns were observed during the previous BA.1 and BA.2 transmission periods compared to the Delta variant [7]. Additionally, during the Omicron BA.1 period, there was a decrease in the severity of symptoms, hospitalizations, and deaths compared to pre-Alpha variants and the displaced Delta variant [24,25]. This difference could be due to the high prior exposure [13], changes in health-seeking behavior, or intrinsic differences between viral lineages. While PCR tests were useful in identifying cases during epidemic SARS-CoV-2 waves, they may not be affordable for community-based surveys, particularly in resource-limited settings. Therefore, it is important to update diagnostic algorithms that consider the presence and combination of symptoms associated with the emergence of new variants.

We found some evidence that immune status was linked to the risk of RT-PCR-detected infection in this population. Individuals who were first infected before the first round of surveys (before November 2020) had a reduced risk of infection. As these people had the greatest opportunity to acquire multiple infections, our results suggest that people who were frequently exposed to SARS-CoV-2 may accumulate protective immunity from multiple prior exposures [19,^26^,27]. Low rates of reporting to national surveillance systems over time mean that cohort studies will become increasingly relied upon to understand immunity to SARS-CoV-2. Such studies should measure immune status, exposure history, and detect incident infections. Assessing COVID-19 transmission through serosurveys can be challenging for open cohorts that may face issues such as loss to follow-up and incomplete registration. Additionally, the presence of vaccines can complicate the interpretation of serological results as they may reflect either infection or vaccination. Here, we use novel methods to integrate PCR-confirmed infections with Ct values to reconstruct the dynamics of infection in this cohort. Due to the challenge of identifying cases through passive surveillance, future studies, including ours, will need to integrate multiple sources of information to characterize the dynamics of infections in populations. We identified a high secondary transmission rate of 31.3% (95% CI 22.2-42.1). While there are no epidemiological studies that confirm the increased infectiousness of the Omicron BQ.1 variant, we used insights from previous variants, such as BA.1 and BA.2, to contextualize our findings [28,29]. It has been reported that the Omicron variant is associated with a ∼50% secondary household transmission [29,30]. The high attack rate observed in our study underscores the urgent need to implement prevention measures in addition to vaccine campaigns to limit transmission.

We acknowledge the limitations in our study. Firstly, the study was conducted during the peak of the outbreak, which may limit our ability to fully characterize the outbreak. Although we estimated cumulative incidence, the uncertainty during the pre-study recruitment period is reflected in the wide 95% credible interval during this period, and our estimate relied on a small number of studies measuring RT-PCR positivity over time following an Omicron infection. Secondly, as described above there was likely misclassification in our identification of prior SARS-CoV-2 exposure using previous serosurveys. Moreover, the use of RT-PCR positivity as the outcome of interest in our regression analysis likely induced misclassification of the outcome of interest (i.e., infection during the outbreak). Thirdly, self-reported data were used to evaluate symptoms, which may have introduced recall bias. Finally, our assumption that all secondary cases within a household were infected by the primary case in the SAR analysis was a simplification and did not account for infections acquired outside of the household. Additionally, as a prevalence survey study, our estimates of incidence outside the study period were moderately sensitive to model assumptions in a sensitivity analysis. Finally, we assumed that symptomatic and asymptomatic individuals had the same Ct distribution following infection, which may have biased our estimate of cumulative incidence. The direction of bias depends on which features of the Ct curve differ by symptom status.

Our findings emphasize the importance of monitoring new variants and their clinical outcomes during the ongoing COVID-19 pandemic. Utilization of new tools, such as mathematical modeling and phylogenetic analysis can improve outbreak characterization and allow for continued monitoring of incidence as the COVID-19 outbreak continues.

## Declarations of competing interest

A.I.K serves as an expert panel member for Reckitt Global Hygiene Institute, scientific advisory board member for Revelar Biotherapeutics and a consultant for Tata Medical and Diagnostics and Regeneron Pharmaceuticals, and has received grants from Merck, Regeneron Pharmaceuticals and Tata Medical and Diagnostics for research related to COVID-19, all of which are outside the scope of the submitted work. D.A.T.C. has received a grant from Merck for research unrelated to COVID-19, outside of the scope of this work. Other authors declare no competing interests.

## Appendix Supplementary materials

Image, application 1
