# Corrigendum to “Extensive transmission of SARS-CoV-2 BQ.1* variant in a population with high levels of hybrid immunity: A prevalence survey” [International Journal of Infectious Diseases 139 (2024) 159-167]

**DOI:** 10.1016/j.ijid.2024.02.007

**Published:** 2024-04

**Authors:** Juan P. Aguilar Ticona, Meng Xiao, Dan Li, Jr. Nivison Nery, Matt Hitchings, Emilia M.M. Andrade Belitardo, Mariam O. Fofana, Renato Victoriano, Jaqueline S. Cruz, Laise de Moraes, Icaro Morais Strobel, Jessica Jesus Silva, Ananias Sena do Aragão Filho, Guilherme S. Ribeiro, Mitermayer G. Reis, Federico Costa, Ricardo Khouri, Albert I. Ko, Derek A.T. Cummings

**Affiliations:** 1 Instituto de Saúde Coletiva, Universidade Federal da Bahia, Salvador, Brazil; 2 Instituto Gonçalo Moniz, Fundação Oswaldo Cruz, Ministério da Saúde, Salvador, Brazil; 3 Department of Epidemiology of Microbial Diseases, Yale School of Public Health, New Haven, United States; 4 Department of Laboratory Medicine, State Key Laboratory of Complex Severe and Rare Diseases, Peking Union Medical College Hospital, Chinese Academy of Medical Sciences and Peking Union Medical College, Beijing, China; 5 Public Health Emergency Center, Chinese Center for Disease Control and Prevention, Beijing, China; 6 Department of Biostatistics, University of Florida, Gainesville, United States; 7 Faculdade de Medicina da Bahia, Universidade Federal da Bahia, Salvador, Brazil; 8 Department of Biology, University of Florida, Gainesville, United States; 9 Emerging Pathogens Institute, University of Florida, Gainesville, United States

The authors regret the funding information included in the final version of the article is incorrect. The correct funding information is:

Research reported in this publication was supported by the National Institute Of Allergy And Infectious Diseases of the National Institutes of Health under Award Numbers R01 AI174105 and R01 AI121207 to A.I.K. and T32AI007517 to M.O.F. (https://www.nih.gov). This work also was supported by grants from the UK Medical Research Council (https://mrc.ukri.org;MR/T029781/1 to F.C.), the Wellcome Trust (https://wellcome.org; 102330/Z/13/Z; 218987/Z/19/Z to F.C.), the Bill and Melinda Gates Foundation (https://www.gatesfoundation.org; OPP1211988 to M.G.R. and F.C.), the Conselho Nacional de Desenvolvimento Científico e Tecnológico [Brazilian National Council for Scientific and Technological Development] (https://www.gov.br/cnpq/pt-br; CNPq 311365/2021-3 to G.S.R.), the Fundação de Amparo à Pesquisa do Estado da Bahia [Bahia Foundation for Research Support] (http://www.fapesb.ba.gov.br; FAPESB SUS0019/2021 and PET0022/2016 to G.S.R.), the Brazilian Department of Science and Technology (DECIT) to R.K., the Brazilian Ministry of Health (MoH) to R.K., the Burroughs-Wellcome Fund (https://www.bwfund.org; ASTMH Postdoctoral Fellowship to M.O.F.), the William H. Prusoff Foundation (Postdoctoral Fellowship to M.O.F.), the China Medical Board (CMB) Global Health Leadership Development Program (Fellowship to M. X. and D.L.), a US NSF RAPID award (to M.D.T.H. and D.A.T.C.), the Coordenação de Aperfeiçoamento de Pessoal de Nível Superior – Brasil [Coordination for the Improvement of Higher Education Personnel] (Doctoral scholarship to J.P.A.T., Finance Code 001), the Raj and Indra Nooyi Professorship, the Sendas Family and Beatrice Kleinberg Neuwirth Funds at the Yale School of Public Health (to A.I.K.). This funding source had no role in the design of this study and will not have any role during its execution, analyses, interpretation of the data, decision to publish, or preparation of the manuscript. The content is solely the responsibility of the authors and does not necessarily represent the official views of the National Institutes of Health or of the donors.

The authors would like to apologise for any inconvenience caused.

