# Supplementary material for: Extensive transmission of SARS-CoV-2 BQ.1* variant in a population with high levels of hybrid immunity: A prevalence survey
Source: Int J Infect Dis. 2024 Feb;139:159–67. doi: 10.1016/j.ijid.2023.11.039 (PMC10784150; doi:10.1016/j.ijid.2023.11.039)
Supplement: Supplementary file 1 [file mmc1.docx]

## Extensive transmission of SARS-CoV-2 BQ.1* variant in a population with high levels of hybrid immunity: A prevalence survey

## Supplementary Material 1

**Prior SARS-CoV-2 Exposure Documented in Study Population**

As mentioned in the main text, our study population had a high prior exposure to SARS-CoV-2, which was identified through serosurveys and active case-finding before the present study. Three serosurveys were conducted, showing an increase in seropositivity (tested by SARS-CoV-2 anti-S IgG) among participants (Figure 1D). The interval periods for the serosurveys were November 2020 to February 2021 (Survey 1), from June to October 2021 (Survey 2), and from March to August 2022 (Survey 3). Additionally, our team conducted active case-finding between November 2021 to October 2022 to identify symptomatic SARS-CoV-2 cases and their contacts in the same area (Figure 1E). Field teams visited study households every two weeks to screen residents for symptoms and collect nasal swabs for SARS-CoV-2 molecular diagnostics.

To determine prior exposure for each recruited resident, we considered seroconversion occurring before the first dose of vaccination as evidence of prior exposure. We also used PCR-confirmed infection during active case-finding to identify additional prior exposures. Based on a summary of all available evidence, we assigned the final prior SARS-CoV-2 exposure status for each individual into four classes:

1. "Yes": Individuals with prior exposure identified in Survey 1, 2, or 3, or during active case-finding, regardless of follow-up status in other study periods.

2. "No": Individuals with complete follow-up, where prior exposure was not found in Surveys 1, 2, and 3, and during active case-finding.

3. "Unknown": Individuals with complete follow-up, where seroconversion was observed during Surveys 1 to 3 but after the first dose of vaccination (thus, the seroconversion cannot be attributed to prior exposure or vaccination). Additionally, prior exposure was not found during active case-finding.

4. "Missing": Individuals with incomplete follow-up in Surveys 1 to 3 or during active case-finding, with no evidence of prior exposure identified.

Furthermore, we used SARS-CoV-2 IgG levels as a proxy for the immune response. Subsequently, we employed logistic regression models to investigate the association between documented prior exposure in surveys, active case-finding before the BQ.1 outbreak, and the subsequent risk of SARS-CoV-2 infection, while adjusting for the number of COVID-19 vaccine doses, week of sample collection, and age due to their potential confounding role.

## Molecular diagnosis

- **RNA extraction and RT-qPCR**

Samples were extracted from 200 µL with Quick-DNA/RNA Viral MagBead Kit (Zymo Research, Cat. no. R2141) using KingFisher Flex System (Thermo Fisher Scientific, Cat. no. 5400630).

The detection of SARS-CoV-2 RNA was performed by RT-qPCR using BIOMOL-OneStep/COVID-19 Kit (Instituto de Biologia Molecular do Paraná, ANVISA no. 80780040004), Molecular SARS-CoV-2 Kit EDx (Bio-Manguinhos, ANVISA no.  80142170045) or CDC 2019-nCoV Reverse Transcriptase PCR Assay [1] on a 7500 Real Time PCR System (Applied Biosystems, Cat. no. 4351105) or QuantStudio 5 Real-Time PCR System (Applied Biosystems, Cat. no. A28574). All protocols followed manufacturer’s instructions.

- **NGS library preparation, sequencing and genome assembly**

The libraries were prepared using the COVIDSeq Test (Illumina, catalog numbers 20043675 and 20043137) with the ARTIC V4 or V4.1 primer set as they became available. Equimolar amounts of all libraries were then pooled together. The fragment length distribution was evaluated using the Agilent Bioanalyzer High Sensitivity DNA Kit (Agilent Technologies, catalog number 5067-4626) on the Agilent 2100 Bioanalyzer (Agilent Technologies, catalog number G2939BA). The concentration was determined using the Qubit 1X dsDNA High Sensitivity Assay Kit (Thermo Fisher Scientific, catalog numbers Q33230 or Q33231) on the Qubit 3 Fluorometer (Thermo Fisher Scientific, catalog number Q33216). The library pool was denatured and diluted to a final loading concentration of 8 pM, and then loaded into either the 300-cycle MiSeq Reagent Kit v2 (Illumina, catalog number MS-102-2002) or the 600-cycle MiSeq Reagent Kit v3 (Illumina, catalog number MS-102-3003). The paired-end sequencing was performed using the Illumina MiSeq (Illumina, catalog number SY-410-1003) with a read length of 150 bp. All protocols were carried out following the manufacturer's instructions.

The fastq files generated were submitted to the pipeline defined by Dezordi and colleagues [2] with minor modifications. In brief, the reads were trimmed to remove low-quality base pairs and primers using fastp v0.23.2, [3] the assembly was performed by Burrows-Wheeler Aligner (BWA) v0.7.17 [4] using NCBI GenBank accession no. MN908947.3 as genome reference. The consensus sequences were then masked with “N” at regions with coverage depth <10, and the variant candidates were incorporated into the consensus genome by using iVAR v1.3.1. [5] The assembly statistics were calculated with SAMtools v.1.16.1 (using HTSlib 1.16.1) [6] and Seqtk v1.3-r106 (https://github.com/lh3/seqtk). The sequences generated in this study is available via the GISAID Epi Set identifier EPI_SET_230212yo (doi: 10.55876/gis8.230212yo).

- **Phylogenetic Analysis**

We obtained data for the SARS-CoV-2 Omicron variant from Salvador, Northeast Brazil, Bahia, from the GISAID database [7] for the period between January 1, 2022, and December 31, 2022, using R version 4.2.2 [8] and the GISAIDR package [9]. To ensure the quality of the analyzed data, only genomes greater than 29,000 base pairs with a variant assignment provided by the Phylogenetic Assignment of Named Global Outbreak Lineages (PANGOLIN) [10] were considered (n = 1,240). The complete set of sequences used in the analysis can be accessed via the GISAID Epi Set identifier EPI_SET_230211te.

A multiple sequence alignment was performed using MAFFT version 7.511 with the options --6merpair and –add fragments [11, 12]. Problematic sites were masked with "N" in the alignment [13] and manually reviewed using AliView version 1.28 [14].

The maximum likelihood (ML) phylogenetic analysis was performed using IQ-TREE version 2.2.0.3 [15] under the generalized time-reversible (GTR) model of nucleotide substitution, incorporating empirical base frequencies (+F), a proportion of invariant sites (+I), and gamma rate heterogeneity across sites with 4 categories (+G4). The model was chosen based on the Bayesian Information Criterion by ModelFinder [16] and included 1,000 replicates of ultrafast bootstrapping (--B 1000) and the SH-aLRT branch test (--alrt 1000) [17]. The ML consensus tree was visualized using R version 4.2.2 with the ggtree package [18-20] and Adobe Illustrator CC 2023 (http://www.adobe.com).

**Assessment of the robustness of the sample**

We conducted a sensitivity analysis to assess whether the selected sample might not be representative of the community. Firstly, we used data obtained from the census conducted in the previous survey (Mar 2022 to Aug 2022), during active finding, and in the current study to evaluate the main characteristics associated with participants recruited and not recruited in this study. We observed that the number of residents was associated with participation (<0.001), as identified by the reviewer, as well as participation in the previous survey (<0.001). However, gender, age, previous vaccination, and seropositivity in the previous survey were similar in both populations (Supplementary Table 4).

Then, we evaluated whether the number of residents predicted a positive result in the PCR test for SARS-CoV-2. To do this, we used a logistic regression model and found no association between the number of residents and testing positive for SARS-CoV-2 in the PCR test (Supplementary Table 5). Subsequently, we performed multiple imputation to address missing values in the PCR results using the binary logistic regression method, considering gender, age, participation in the previous survey, and the number of residents in the houses, creating five complete datasets. Then, we used bootstrap sampling to select 1000 samples from each of the 5 datasets, each with 535 participants, as in our study to evaluate the robustness of the sample. With that we calculated the proportions of positive PCR cases in these random samples and the final results show that the obtained proportion of 14.8% positive PCR results used to estimate the incidence in our study , it is included within the expected values for a representative sample of the population, with an expected proportion of 13.9% (95%CI 11.0% - 16.9%) (Supplementary Table 6).

In conclusion, although the number of residents is higher in the selected sample, it is not associated with the results of the PCR test. The sample proved to be robust in determining positivity during the study period in this community.

## Estimation of cumulative incidence

To inform the estimate of daily risk of infection using Ct values and PCR positivity prevalence, the model of Hay et al. [21] assumes a distribution of Ct over time following infection, and a corresponding probability of being detectable. To account for differential dynamics of viral load following Omicron infection compared to earlier variants, we updated the parameters used in the original paper using the results of longitudinal studies conducted among Omicron patients. Specifically Hay et al. [21] found that peak Ct of between 25.0 and 26.2 among individuals infected with Omicron, proliferation time (i.e. time to peak viral load) ranging between 3.3 and 4.3 days, and clearance time (i.e. time to maximum Ct following the peak) rangin gfrom 5.8 to 8.7 days. Therefore, we set the peak Ct at 25, time to peak at 3.8 days, and time to clearance at 7 days. To allow for quicker loss of detection following clearance, we set the probability of loss of detectability to 0.2 per day. With these parameters, the model produced close to 0% PCR positivity at 28 days following infection, consistent with Hay et al. [21] and Boucau et al. [22], and a median duration of PCR positivity of 15 days, consistant with 14.3 days from Kojima et al [23]. Finally, to allow the epidemic curve to have a sharper peak, we relaxed the autocorrelation used in the Gaussian Process model describing incidence over time, using 𝜌 = 0.06.

**Assessment of the robustness of the incidence estimation.**

In brief, the method of Hay et al assumes a distribution of CT values and probability of PCR positivity over time since infection, and uses individual-level PCR results to infer the likely time of infection among PCR positive individuals, and the proportion of individuals ever infected based on the prevalence of PCR positivity. The likelihood of observing a given CT *y* value in a PCR positive individual sampled on day *t* is the probability that they were infected some days *d* previously, multiplied by the probability of having a detectable CT value and of having CT value *y* *d* days later, summed over all possible values of *d*. If π_t-d_ is the probability of being infected *d* days before *t*, φ_d_ is the probability of detectable CT *a* days after infection, and p_d_(y_i_) is the probability of having CT values y_i_ *d* days after infection for an individual with detectable CT, then the likelihood of observing a given CT in a PCR-positive individual is

$$\Pr\left( Y_{i}=y_{i} | \pi_{t-D_{max}},\ldots,\pi_{t-1} \right)=\sum_{d=1}^{D_{max}} p_{d}\left( y_{i} \right)\phi_{d}\pi_{t-d}$$

where D­_max_ is the maximum duration of PCR positivity. Similarly, the probability of an individual not having detectable CT (i.e. being PCR negative or having Y=C_LOD_, the limit of detection of CT) is simply one minus the probability of an individual having detectable CT on the day of sampling, i.e.

$$\Pr\left( Y_{i}=C_{LOD} | \pi_{t-D_{max}},\ldots,\pi_{t-1} \right)=1-\sum_{d=1}^{D_{max}} \phi_{d}\pi_{t-d}$$

The likelihood for the whole sample can be constructed by multiplying together the individual likelihood contributions. The daily probability of infection is modeled using a Gaussian process, such that daily infection probabilities are correlated (see Hay et al Supplementary Material for details).

We performed sensitivity analyses to check the robustness of the cumulative incidence estimate to changes in the CT distribution and PCR positivity probability over time. Specifically, we assumed: longer time to loss of detectability following clearance (consistent with Saade et al. [24] and Luna-Muschi et al. [25]); faster time from peak viral load to viral clearance (4 days vs 7 days); higher peak viral load (peak CT 20 vs. 25); and higher viral load following viral clearance (CT following clearance 35 vs 38) (Supplementary Table 7).

Analysis code for the incidence estimation are available on Github (<https://github.com/mhitchings/BQ1_PCR_CumulativeIncidence>).

**References**

1. Centers for Disease Control and Prevention. CDC 2019-novel coronavirus (2019-nCoV) real-time RT-PCR diagnostic panel. 2020.

2. Dezordi FZ, Neto A, Campos TL, et al. ViralFlow: A Versatile Automated Workflow for SARS-CoV-2 Genome Assembly, Lineage Assignment, Mutations and Intrahost Variant Detection. Viruses **2022**; 14.

3. Chen S, Zhou Y, Chen Y, Gu J. fastp: an ultra-fast all-in-one FASTQ preprocessor. Bioinformatics **2018**; 34:i884-i90.

4. Li H, Durbin R. Fast and accurate long-read alignment with Burrows-Wheeler transform. Bioinformatics **2010**; 26:589-95.

5. Castellano S, Cestari F, Faglioni G, et al. iVar, an Interpretation-Oriented Tool to Manage the Update and Revision of Variant Annotation and Classification. Genes (Basel) **2021**; 12.

6. Danecek P, Bonfield JK, Liddle J, et al. Twelve years of SAMtools and BCFtools. Gigascience **2021**; 10.

7. Khare S, Gurry C, Freitas L, et al. GISAID's Role in Pandemic Response. China CDC Wkly **2021**; 3:1049-51.

8. R Core Team R. R: A language and environment for statistical computing. **2013**.

9. Wirth W, Duchene S. GISAIDR. Zenodo; 2022.

10. O'Toole Á, Scher E, Underwood A, et al. Assignment of epidemiological lineages in an emerging pandemic using the pangolin tool. Virus Evol **2021**; 7:veab064.

11. Katoh K, Misawa K, Kuma K, Miyata T. MAFFT: a novel method for rapid multiple sequence alignment based on fast Fourier transform. Nucleic Acids Res **2002**; 30:3059-66.

12. Katoh K, Standley DM. MAFFT multiple sequence alignment software version 7: improvements in performance and usability. Mol Biol Evol **2013**; 30:772-80.

13. Turakhia Y, De Maio N, Thornlow B, et al. Stability of SARS-CoV-2 phylogenies. PLoS Genet **2020**; 16:e1009175.

14. Larsson A. AliView: a fast and lightweight alignment viewer and editor for large datasets. Bioinformatics **2014**; 30:3276-8.

15. Minh BQ, Schmidt HA, Chernomor O, et al. IQ-TREE 2: New Models and Efficient Methods for Phylogenetic Inference in the Genomic Era. Mol Biol Evol **2020**; 37:1530-4.

16. Kalyaanamoorthy S, Minh BQ, Wong TKF, von Haeseler A, Jermiin LS. ModelFinder: fast model selection for accurate phylogenetic estimates. Nat Methods **2017**; 14:587-9.

17. Hoang DT, Chernomor O, von Haeseler A, Minh BQ, Vinh LS. UFBoot2: Improving the Ultrafast Bootstrap Approximation. Mol Biol Evol **2018**; 35:518-22.

18. Yu G, Smith DK, Zhu H, Guan Y, Lam TTY. ggtree: an R package for visualization and annotation of phylogenetic trees with their covariates and other associated data. Methods in Ecology and Evolution **2017**; 8:28-36.

19. Yu G, Lam TT, Zhu H, Guan Y. Two Methods for Mapping and Visualizing Associated Data on Phylogeny Using Ggtree. Mol Biol Evol **2018**; 35:3041-3.

20. Yu G. Using ggtree to Visualize Data on Tree-Like Structures. Curr Protoc Bioinformatics **2020**; 69:e96.

21. Hay JA, Kissler SM, Fauver JR, et al. Quantifying the impact of immune history and variant on SARS-CoV-2 viral kinetics and infection rebound: A retrospective cohort study. eLife **2022**; 11:e81849.

22. Boucau J, Marino C, Regan J, et al. Duration of Shedding of Culturable Virus in SARS-CoV-2 Omicron (BA.1) Infection. New England Journal of Medicine **2022**; 387:275-7.

23. Kojima N, Roshani A, Klausner JD. Duration of COVID-19 PCR positivity for Omicron vs earlier variants. J Clin Virol Plus **2022**; 2:100085.

24. Saade C, Brengel-Pesce K, Gaymard A, et al. Dynamics of viral shedding during ancestral or Omicron BA.1 SARS-CoV-2 infection and enhancement of pre-existing immunity during breakthrough infections. Emerg Microbes Infect **2022**; 11:2423-32.

25. Luna-Muschi A, Noguera SV, Borges IC, et al. Characterization of Severe Acute Respiratory Syndrome Coronavirus 2 Omicron Variant Shedding and Predictors of Viral Culture Positivity on Vaccinated Healthcare Workers With Mild Coronavirus Disease 2019. The Journal of Infectious Diseases **2022**; 226:1726-30.


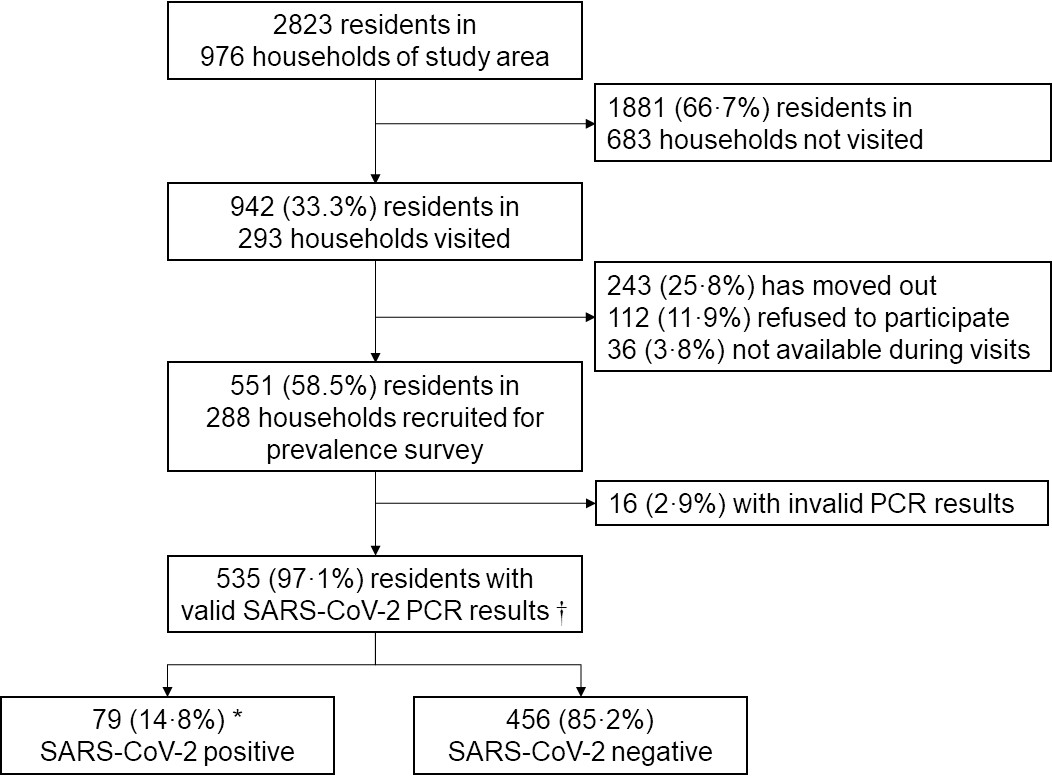


**Supplementary Figure 1.** Study flowchart. * Four only defined by rapid antigen test (RAT), among them two were RT-PCR negative, one RT-PCR intermediate and one RT-PCR not completed. † Invalid PCR results indicated samples failed to amplify internal controls.

**
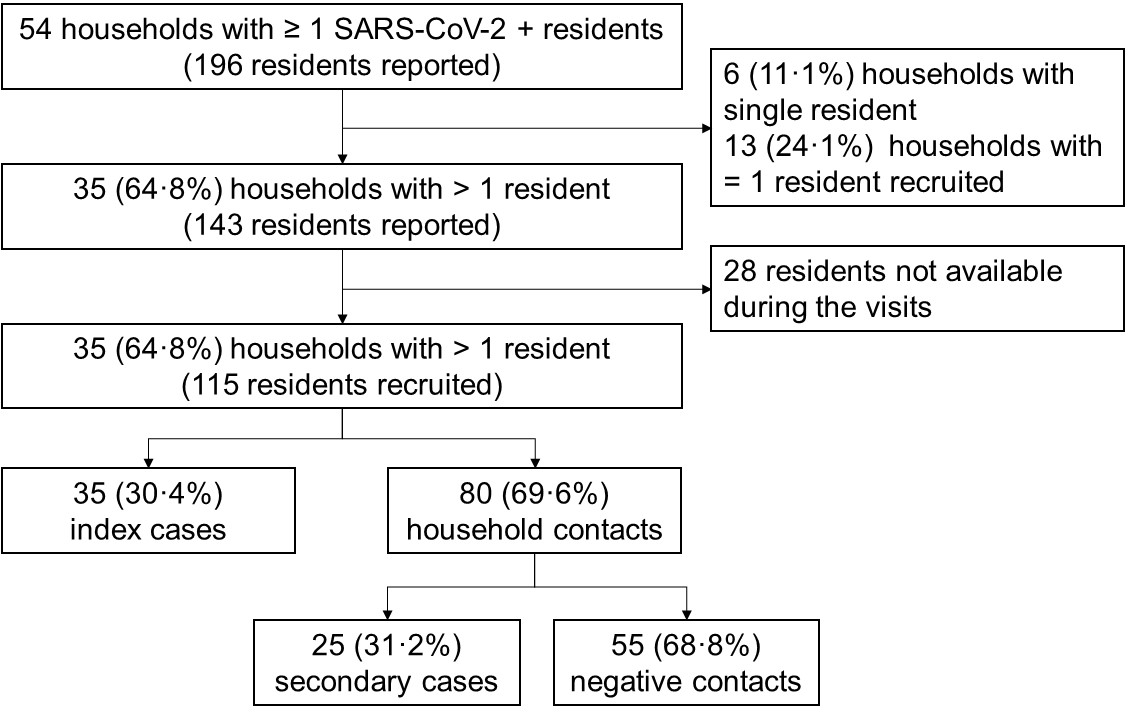
**

**Supplementary Figure 2.** Flowchart of households selected for estimating secondary attack rate. The index case was defined as the individual with the earliest positive test or onset date for symptoms in the household. If two members had the same earliest date for positive test and symptoms, they were considered co-index cases. If infection order could not be determined for any household member, that household was excluded from the SAR analyses (co-index cases).


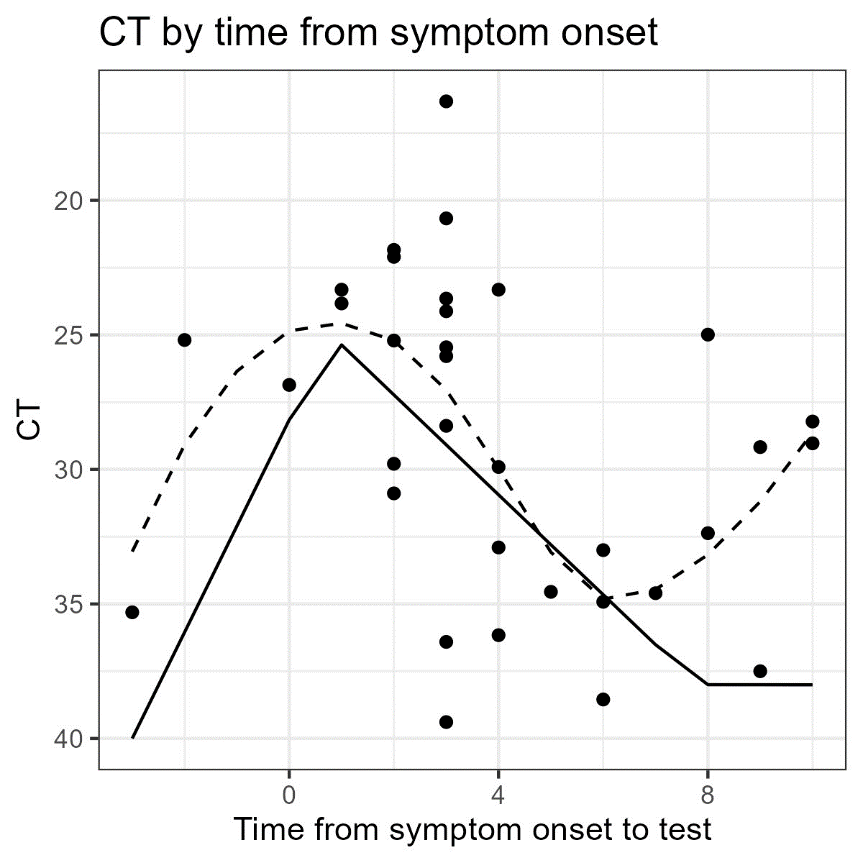


**Supplementary Figure 3.** Ct value over time from symptom onset for individuals with positive PCR test and recorded symptom onset date, with a LOESS smoother fit to the data (dotted line) and the assumed Ct distribution over time assumed by the model (solid line), assuming three days from infection to symptom onset


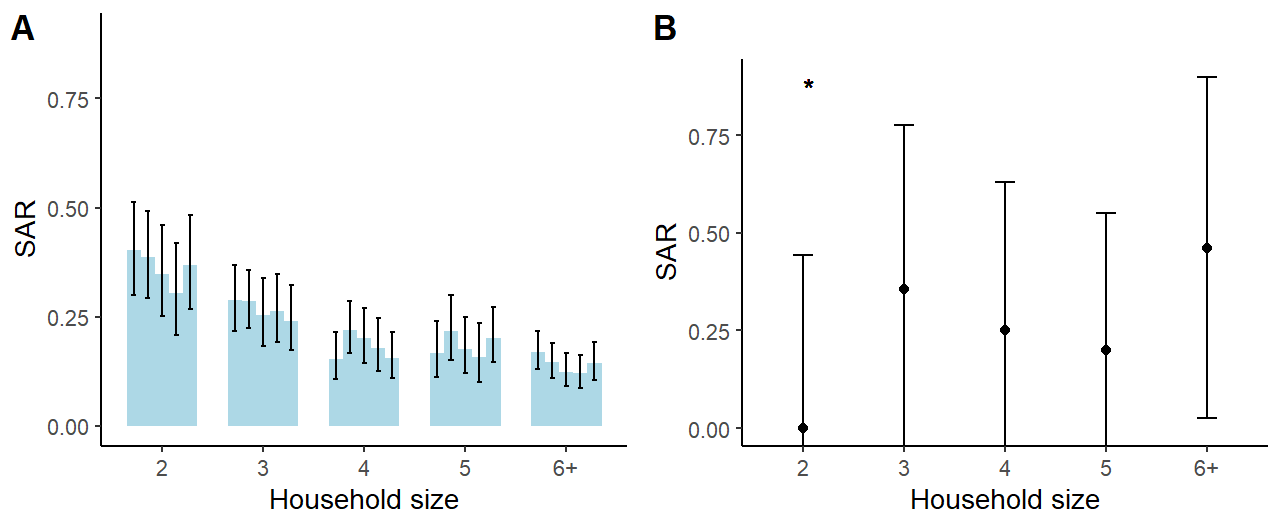


**Supplementary Figure 4.** Secondary Attack Rate distributed by number of residents in the household A) data obtained by multiple imputation and B) sample study

* 95%CI for a binomial proportion using the Agresti-Coull method

**Supplementary Table 1. Household secondary attack rate (SAR) and risk factors.**

| Secondary cases characteristic | No. secondary cases /No. contacts | SAR (%) (95% CI) | RR (95%CI) |
| --- | --- | --- | --- |
| All | 25/80 | 31.3 (22.2-42.1) |  |
| Age group |  |  |  |
| <18 | 15/34 | 44.1 (28.9-60.6) | 2.03 (1.04-3.95) |
| ≥18 | 10/46 | 21.7 (12.3-35.6) | 1 [Reference] |
| 18 - 35 | 6/24 | 25.0 (12.0-44.9) |  |
| 36 - 59 | 2/18 | 11.1 (3.1-32.8) |  |
| ≥60 | 2/4 | 50.0 (15.0-85.0) |  |
| Sex |  |  |  |
| Female | 15/45 | 33.3 (21.4-47.9） | 1.16 (0.60-2.27) |
| Male | 10/35 | 28.6 (16.3-45.1） | 1 [Reference] |
| Vaccine dose(s) received |  |  |  |
| ≥3 doses | 10/26 | 38.5 (22.4-57.5) | 0.87 (0.36-2.08) |
| 2 doses | 4/27 | 14.8 (5.9-32.5) | 0.33 (0.10-1.07) |
| 1 dose | 7/18 | 38.9 (20.3-61.4) | 0.63 (0.24-1.65) |
| 0 dose | 4/9 | 44.4 (18.9-73.3) | 1 [Reference] |

**Supplementary Table 2.** **Prior SARS-CoV-2 exposure during the major cohort study.**

| Prior exposure | No. (%) | | p-value |
| --- | --- | --- | --- |
|  | SARS-CoV-2  positive  n = 79 | SARS-CoV-2 negative  n = 456 |  |
| Seropositive in Survey 1^a^ |  |  | 0.157 |
| Yes | 17 (10.4%) | 146 (89.6%) |  |
| No | 36 (17.4%) | 171 (82.6%) |  |
| Incomplete follow-up^b^ | 26 (15.8%) | 139 (84.2%) |  |
| Seroconversion in Survey 2 and not previously vaccinated^c^ |  |  | 0.047 |
| Yes | 5 (8.1%) | 57 (91.9%) |  |
| No | 17 (17.7%) | 79 (82.3%) |  |
| Unknown | 31 (12.3%) | 222 (87.7%) |  |
| Incomplete follow-up^b^ | 26 (21.0%) | 98 (79.0%) |  |
| Seroconversion in Survey 3 and not previously vaccinated^c^ |  |  | 0.083 |
| Yes | 4 (11.1%) | 32 (88.9%) |  |
| No | 0 (0.0%) | 6 (100.0%) |  |
| Unknown | 55 (13.4%) | 354 (86.6%) |  |
| Incomplete follow-up^b^ | 20 (23.8%) | 64 (76.2%) |  |
| PCR-confirmed infection during active case finding |  |  | 0.957 |
| Yes | 7 (15.9%) | 37 (84.1%) |  |
| No | 70 (14.6%) | 409 (85.4%) |  |
| Incomplete follow-up^b^ | 2 (16.7%) | 10 (83.3%) |  |
| Prior documented SARS-CoV-2 exposure^c^ |  |  | 0.018 |
| Yes | 31 (10.7%) | 258 (89.3%) |  |
| No | 0 (0.0%) | 6 (100.0%) |  |
| Unknown | 27 (21.3%) | 100 (78.7%) |  |
| Incomplete follow-up | 21 (18.6%) | 92 (81.4%) |  |
| Prior documented SARS-CoV-2 exposure or vaccination^c^ |  |  | >0.999 |
| Yes | 77 (14.9%) | 441 (85.1%) |  |
| No | 2 (11.8%) | 15 (88.2%) |  |

^a^ As SARS-CoV-2 vaccines were not available to public until the end of Survey 1, any seropositive observed in Survey 1 was considered due to SARS-CoV-2 infection.

^b^ Incomplete follow-up in Surveys 1-3 indicates individuals were not recruited in corresponding surveys. Incomplete follow-up in active case finding indicates individuals were not visited at least once during the complete study period.

^c^ See definitions of prior SARS-CoV-2 exposure classifications in Supplementary Material 1.

**Supplementary Table 3. Factors associated with SARS-CoV-2 positive result.**

|  | n | Positive | Negative | Odds Ratio | 95%CI | p |
| --- | --- | --- | --- | --- | --- | --- |
| Model 1 |  |  |  |  |  |  |
| Seropositive in Survey 1, n (%) | 350 | 17 (21.5%) | 146 (32.0%) | 0.50 | 0.25 – 0.97 | 0.045 |
| Model 2 |  |  |  |  |  |  |
| SARS-CoV-2 IgG OD value in Survey 3, mean (SD) | 416 | 2.79 (0.622) | 2.76 (0.760) | 1.15 | 0.71 – 1.91 | 0.571 |
| Model 3 |  |  |  |  |  |  |
| Seropositive in Survey 1, n (%) | 308 | 16 (36.4%) | 131 (49.6%) | 0.52 | 0.24 – 1.07 | 0.080 |
| SARS-CoV-2 IgG OD value in Survey 3, mean (SD) | 308 | 2.89 (0.503) | 2.82 (0.714) | 1.36 | 0.75 – 2.53 | 0.317 |
| Model 4 |  |  |  |  |  |  |
| Seropositive in Survey 1, n (%) | 350 | 17 (33.3%) | 146 (48.8%) | 0.50 | 0.25 – 0.98 | 0.046 |
| PCR-confirmed infection in active case finding, n (%) | 350 | 5 (9.8%) | 27 (9.0%) | 1.06 | 0.32 – 3.03 | 0.915 |

All models were adjusted by age, the number of vaccine doses and the week of sample collection.

**Supplementary Table 4. Characteristics of recruited and non-recruited Pau da Lima residents**

| **Characteristic** | **Responses** | **Recruited n = 942** | **Non-recruited n = 1881** | **p-value** |
| --- | --- | --- | --- | --- |
| Available information, n (%)* | 2,823 |  |  | **<0.001** |
| Yes |  | 920 (97.7) | 1740 (92.9) |  |
| No |  | 22 (2.3) | 141 (8.1) |  |
| Age in years | 2,658 | 32 (18) | 32 (19) | 0.74 |
| Sex, n (%) | 2,660 |  |  | 0.31 |
| Female |  | 494 (54) | 976 (56) |  |
| Male |  | 423 (46) | 767 (44) |  |
| Number of residents | 2,660 | 3.94 (1.99) | 3.39 (1.87) | **<0.001** |
| Vaccination, n (%) | 2,105 |  |  | 0.60 |
| Yes |  | 647 (85) | 1,160 (86) |  |
| No |  | 112 (15) | 186 (14) |  |
| Participate in previous survey L47, n (%) | 2,655 |  |  | **<0.001** |
| Yes |  | 685 (75) | 908 (52) |  |
| No |  | 229 (25) | 833 (48) |  |
| IgG SARS-CoV-2 result in the previous survey, n (%) | 1,760 |  |  | 0.25 |
| Positive |  | 605 (98) | 1,130 (99) |  |
| Negative |  | 12 (1.9) | 13 (1.1) |  |
| BQ1 survey PCR result, n (%) | 2,660 |  |  | **<0.001** |
| Positive |  | 79 (8.6) | 0 (0) |  |
| Negative |  | 456 (49) | 0 (0) |  |
| Invalid PCR |  | 16 (1.7) | 0 (0) |  |
| Non recruited |  | 369 (40) | 1,740 (100) |  |

* Data obtained through the population census, previous surveys, active case finding, and during the completed survey in the cohort study

**Supplementary Table 5. Logistic regression to evaluate the effect of the number of residents in the PCR SARS-CoV-2 positive result.**

| **Characteristics** | **Odds Ratios** | **95%CI** | **p** |
| --- | --- | --- | --- |
| Intercept | 0.19 | 0.06 – 0.60 | 0.006 |
| Number of residents | 1.09 | 0.96 – 1.22 | 0.184 |
| Age in years | 1.00 | 0.99 – 1.01 | 0.957 |
| Male | 0.82 | 0.49 – 1.35 | 0.445 |
| Vaccinated | 1.33 | 0.66 – 2.89 | 0.450 |
| Participate in previous survey L47 | 0.49 | 0.27 – 0.92 | 0.021 |
| Observations | 535 | | |

**Supplementary Table 6. Sensitivity analysis employing multiple imputation techniques to assess the sample's robustness in evaluating the proportion of PCR SARS-CoV-2 positive result among a sample of 535 participants**

|  | **Proportion of PCR SARS-CoV-2 +** | **95%CI** | |
| --- | --- | --- | --- |
| dataset 1 | 13.5% | 10.5% | 16.4% |
| dataset 2 | 16.7% | 13.6% | 19.8% |
| dataset 3 | 12.7% | 9.7% | 15.5% |
| dataset 4 | 13.2% | 10.3% | 16.1% |
| dataset 5 | 13.7% | 10.7% | 16.5% |
| Pooled proportion | 13.9% | 11.0% | 16.9% |

**Supplementary Table 7. Estimated cumulative incidence from October 19 to December 22, 2022, adjusting parameters relating to PCR CT distribution and probability of PCR positivity over time following infection**

| **Model** | **Estimated CI (95% CrI)** | **Estimated day of peak incidence (95% CrI)** |
| --- | --- | --- |
| Base model | 56% (36%, 88%) | Nov 17 (Nov 9, Nov 21) |
| Longer time to loss of detectability (daily probability of loss of detectability following clearance = 0.1) | **49%** (33%, 76%) | Nov 16 (Nov 9, Nov 21) |
| Faster time to clearance (time from peak to maximum CT = 4 days) | **62%** (40%, 94%) | Nov 17 (Nov 6, Nov 21) |
| Lower peak CT (peak CT = 20) | 54% (34%, 86%) | Nov 17 (Nov 5, Nov 21) |
| Lower CT after clearance (CT=35) | 53% (34%, 85%) | Nov 16 (Nov 4, Nov 20) |

In bold, the maximum and minimum values.

**Supplementary Table 8. Sensitivity analysis employing multiple imputation techniques to assess the household secondary attack rate**

|  | SAR | 95%CI | |
| --- | --- | --- | --- |
| dataset 1 | 20.9% | 13.9% | 28.7% |
| dataset 2 | 22.4% | 14.8% | 31.3% |
| dataset 3 | 19.1% | 13.0% | 27.0% |
| dataset 4 | 17.7% | 11.3% | 26.1% |
| dataset 5 | 19.3% | 13.0% | 25.2% |
| Pooled | 20.5% | 13.2% | 27.7% |
